# A combined strategy for quantitative trait loci detection by genome-wide association

**DOI:** 10.1186/1753-6561-3-s1-s6

**Published:** 2009-02-23

**Authors:** Alex C Lam, Joseph Powell, Wen-Hua Wei, Dirk-Jan de Koning, Chris S Haley

**Affiliations:** 1 Division of Genetics and Genomics, Roslin Institute and Royal (Dick) School of Veterinary Studies, University of Edinburgh, Roslin, Midlothian, EH25 9PS, UK; 2 Institute of Evolutionary Biology, Ashworth Laboratories, Kings Buildings, University of Edinburgh, Edinburgh, EH9 3JT, UK; 3 MRC Human Genetics Unit, Western General Hospital, Edinburgh EH4 2XU, UK

## Abstract

**Background:**

We applied a range of genome-wide association (GWA) methods to map quantitative trait loci (QTL) in the simulated dataset provided by the 12^th ^QTLMAS workshop in order to derive an effective strategy.

**Results:**

A variance component linkage analysis revealed QTLs but with low resolution. Three single-marker based GWA methods were then applied: Transmission Disequilibrium Test and single marker regression, fitting an additive model or a genotype model, on phenotypes pre-corrected for pedigree and fixed effects. These methods detected QTL positions with high concordance to each other and with greater refinement of the linkage signals. Further multiple-marker and haplotype analyses confirmed the results with higher significance. Two-locus interaction analysis detected two epistatic pairs of markers that were not significant by marginal effects. Overall, using stringent Bonferroni thresholds we identified 9 additive QTL and 2 epistatic interactions, which together explained about 12.3% of the corrected phenotypic variance.

**Conclusion:**

The combination of methods that are robust against population stratification, like QTDT, with flexible linear models that take account of the family structure provided consistent results. Extensive simulations are still required to determine appropriate thresholds for more advanced model including epistasis.

## Background

With recent advances in genotyping technology, high density marker maps are becoming commonly used to map the genetic loci controlling complex trait variation. Most large-scale genome-wide association (GWA) studies published to date, such as those conducted by the Wellcome Trust Case Control Consortium [1], used case-control designs with individuals selected to be unrelated. New methods such as GRAMMAR [2] allow effective and robust GWA studies on general pedigreed populations like the simulated data provided by the 12^th ^QTL-MAS workshop . Here we describe a comprehensive set of GWA analyses to detect quantitative trait loci (QTL) in the simulated population in order to compare the commonly used methods of linkage, transmission disequilibrium test (TDT), and single marker association with more experimental models including multiple marker and haplotype associations and epistasis. Based on the comparisons we aim to derive a generic strategy for GWA studies on general pedigreed populations.

## Methods

The simulated population consists of 4665 individuals across four generations. From the first generation, 15 sires, each mated 10 dams that produced 10 progeny per full-sib family. Each individual was phenotyped for one continuous trait and genotyped with 6,000 Single Nucleotide Polymorphism (SNP) markers without missing values. The SNP data were phased and treated as evenly spaced across six 100 cM chromosomes.

Haploview [3] was used to estimate minor allele frequencies (MAF) and linkage disequilibrium (LD) within a 20 marker window. We also estimated descriptive statistics including the total variance and heritability and examined for normality. Eighty four SNPs with MAF below 0.1% were excluded from further analyses. The LOD score of 3, equivalent to the *P*-value of 2*10-4, was used as the threshold for linkage analyses. For all single-QTL association studies, Bonferroni correction of 5916 tests was used to derive the 5% genome-wide threshold resulting in the nominal *P*-value of 8.45*10^-6^, or 5.08 in the -log_10_(*P*) transformation (logP). That threshold was used consistently across the GWA analyses in this study to detect markers that significant by their marginal effects (denoted as qSNP). Although the Bonferroni correction is known for being too conservative, it is easily implemented and much less computer-intensive than permutation tests. Furthermore, the resulting *P*-value threshold is in line with many published GWA studies.

**Figure 1:**
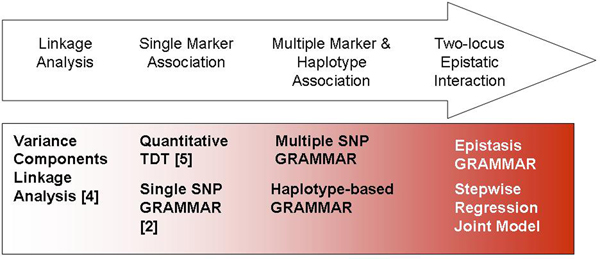
**A flow diagram of the methods used**.

Figure 1 shows the analysis framework used in this study; the methods are described in the following sections.

### QTL analyses based on transmission of alleles within full-sib families

The pedigree was divided into 450 nuclear families. At first, a variance components linkage analysis [4] was used to evaluate the significance of the additive genetic variance component. Then, we performed genome-wide association using two methods implemented in the software QTDT [5]. These methods model the allelic means for a test of association having accounted for the sib-pair covariance structure. The first method is the *de facto *QTDT, where the allelic association is evaluated within the nuclear families only. Using the within-family component solely in evaluating the allelic association is robust to admixture in the population. Secondly, without partitioning the mean effect of a locus into the between- and within-family components, testing of the total association was also carried out. Such a test is not a TDT, although it is implemented in the QTDT software, and it is a less conservative test compared to QTDT when population stratification can be ignored.

### Single SNP GRAMMAR

The first stage of GRAMMAR [2] was adopted to correct the phenotype for pedigree and fixed effects using ASREML [6]. The mixed model fitted a random effect of pedigree and fixed effects of sex and generation. The residuals obtained for each individual were used as the corrected trait in the GWA analyses below. The single marker association was modelled in two ways: fitting the additive allelic effect as a covariate or the genotype classes as fixed factors where both additive and dominance effects can be estimated.

### Multiple-markers and haplotype analysis

Using the pre-corrected phenotypic values, we evaluated the joint effect of multiple SNPs within a three marker sliding window. Markers were fitted as individual linear covariates within a multiple regression framework to test for their joint association. Using the same sliding window method haplotypes were estimated from 3 adjacent SNPs with the software "haplo.stats" in R [7]. A progressive insertion EM algorithm determines haplotype frequencies which are then used to test for association with a score statistic [8]. A three marker window was chosen to reduce computational time for the haplotype method and applied to both analyses for consistency. Further work is required to investigate the effect of alternative marker window sizes on power to detect QTL.

### Two-locus interaction analysis

A four-stage approach was used to analyse epistasis based on the pre-corrected phenotypes where SNP genotypes were fitted as fixed factors: 1) single SNP regression to identify qSNPs (see above); 2) detect qSNP × qSNP pairs [9]; 3) detect qSNP × non-qSNP pairs; 4) detect non-qSNP × non-qSNP pairs. Nested tests were used to identify significant epistatic pairs; the first test compares the full model (y = μ+SNP_1_+SNP_2_+SNP_1 _*SNP_2_+e) with the NULL model (y = μ+e); the second test compares the full model with the two-SNP model (y = μ+SNP_1_+SNP_2_+e) (i.e. epistasis test). Only pairs that were significant for the first test enter the epistasis test. When either SNP_1 _or SNP_2 _is a qSNP, the first test is changed to ensure the full model is better than the single SNP model (y = μ+qSNP +e) before the epistasis test. When both SNP_1 _and SNP_2 _are qSNPs, only the epistasis test is needed. To avoid spurious interactions between closely located SNPs an arbitrary minimum distance of 10 cM was applied to any interacting SNP pairs.

The 5% genome wide thresholds were derived for the nested tests using Bonferroni correction based on the number of tests (assuming independent tests). Suppose ***K ***qSNPs are identified from ***N ***available SNPs in stage one, the number of the first tests is in the order of ***N***^2^, K*N and ***K***^2 ^for the non-qSNP × non-qSNP, qSNP × non-qSNP and qSNP × qSNP pairs, respectively. The number of pairs that are significant for the first test is used to derive the 5% genome wide threshold for the epistasis tests.

### Results integration

Forward linear regression was used to integrate the mapping results in the order of a) qSNP × qSNP pairs; b) qSNP × non-qSNP pairs; c) non-qSNP × non-qSNP pairs and d) qSNPs using their corresponding thresholds. The epistatic pairs were fitted first because they also capture the marginal effects of the qSNPs that were involved in these pairs. QTL (pairs) were included in the model in order of decreasing significance. QTL or QTL pairs were dropped when their individual P value was smaller than their corresponding threshold.

## Results and discussion

### Descriptive statistics

The uncorrected trait data was approximately normally distributed using a Q-Q plot. It ranged from -5.36 to 8.67, with mean and standard deviation of 1.36 and 2.10 respectively. No differences in the distribution were observed due to sex. The estimated heritability was 29.6%. The LD between adjacent SNP pairs was generally low; the mean r^2 ^between adjacent markers was 0.2 and decreased linearly with map distance. However, much higher LD was observed when looking at all pairwise r^2 ^values within a 20 marker window – the mean maximum r^2 ^between two SNPs was 0.62.

### Linkage and association tests using QTDT

**Figure 2:**
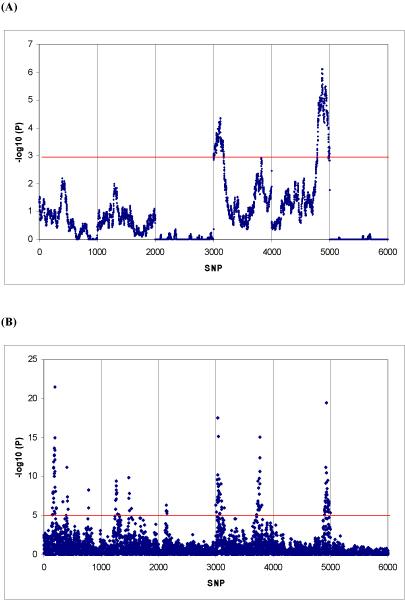
**(A) Linkage and (B) association profiles**. **(A) **The linkage profile generated from a variance component linkage analysis. Y-axis shows the -LOG_10 _transformed p-values and the x-axis shows the positions of SNPs along the genome. Vertical lines denote the chromosome boundaries. The significance threshold of LOD = 3 is shown by the red line. **(B)** The association results produced by the single marker additive model using GRAMMAR. -LOG_10 _transformed p values are given for each marker position. The genome-wide significance threshold (p < 0.05) is shown with the red line.

Linkage analysis revealed strong evidence of QTL on chromosomes 4 and 5 (Figure 2A). Tests for within-family association pointed to QTL locations on chromosomes 1–5 In total 194 SNPs were significant. Since we have observed high LD between non-adjacent SNPs, we were concerned that multiple SNPs could be called significant simply due to LD. As a conservative measure, we grouped the significant SNPs into 9 QTL peaks. These peaks were defined as the most significant marker and its neighbouring significant SNPs being at least 10 cM from the next group of significant SNPs. Although the tests for total association gave more significant p-values (results not shown), the results of the QTDT tests for within-family association are considered more robust. The reason is that the way the nuclear families were created from the entire pedigree would have introduced some degree of population substructure. Testing for total association ignores the fact that some individuals sired multiple families and thus, the nuclear families are not independent.

### Single-marker association using GRAMMAR

The additive single SNP analysis identified 9 QTL peaks by visual inspection (Figure 2B). In total 133 individual markers were above the Bonferroni corrected significance threshold. The Genotypic model identified the same 9 QTL peaks but with a total of 108 significant SNPs. Compared to the linkage results, GWA detected more significant SNP signals on chromosomes 1–3 and was consequently deemed more powerful.

Because the single-marker GRAMMAR identified the same peaks as QTDT, we concluded that the benefit of the robustness to admixture of QTDT is negligible in the current dataset. GRAMMAR is more flexible for modelling epistasis and performing the joint analysis. Therefore, GRAMMAR was chosen over QTDT for the subsequent analyses that follow.

### Multiple-marker and haplotype association

The multiple marker analysis identified a total of 9 QTL peaks in the same locations as those identified by the single-marker analyses. Overall, the multiple marker method identified a total of 320 individual significant SNPs. The Haplotype analysis identified the same 9 QTL peaks as the multiple-marker method with a total of 338 significant SNPs.

### Two-locus interaction

The 108 qSNPs identified in Stage one were used for epistasis analyses. The thresholds for the epistatic analyses were as follows: For the 108 qSNPs, the logP threshold for their pair-wise analyses was 5.08. For the interaction between qSNPs and non-qSNPs the logP threshold was 7.01 against the H_0 _of only a single qSNP effect. Following this test, 3040 pairs were significant and the Bonferroni corrected logP threshold for the epistasis test was 4.78. For the pairwise analysis of non-qSNP pairs the logP threshold against H_0 _of no QTL effect was 8.51. Following this test, 99634 pairs were significant and the Bonferroni correct LogP threshold for the epsitais test was 6.3. Two significant non-qSNP × non-qSNP pairs were detected: 1) SNPs 540 and 3219 on chromosomes 1 and 4, respectively; 2) SNPs 1257 and 3689 on chromosomes 2 and 4 respectively. It was interesting that the adjacent SNPs of 1257 (i.e. 1256 and 1258) were both qSNPs with an r^2 ^of 0.75 whereas the LD between 1257 and 1256 (0.26) or 1258 (0.46) was much lower. No significant interactions with any of the 108 qSNPs were found.

### Result integration and overall discussion

**Figure 3:**
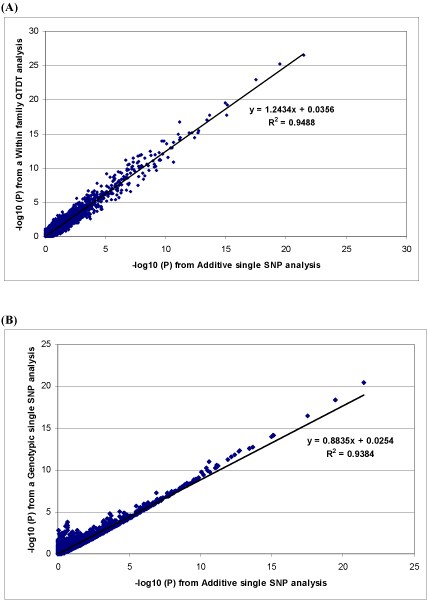
**Comparison of single-marker methods**. **(A) Additive single marker method compared to the within family QTDT. (B) Additive single marker method compared to the genotypic single marker method**. The scatter plots show high correlations between the different single-marker methods used, despite the difference in the magnitude of p-values.

**Figure 4:**
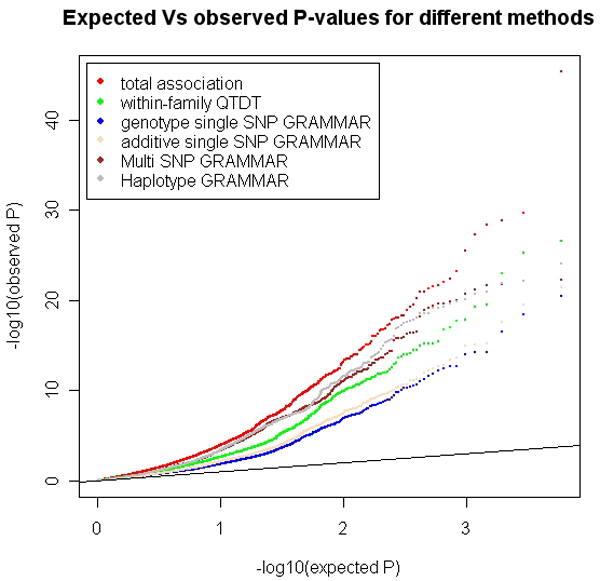
**Power comparison of different single-marker methods**. The Q-Q plot of all methods used shows that the all methods are well-powered to detect QTL. There is an increase in power for the methods using multiple markers over single marker GRAMMAR methods.

The 108 qSNPs and the two epistatic pairs were integrated using the forward selection regression method to remove possible redundancy among those SNPs. The two epistatic pairs and 9 qSNPs remained significant in the final model and jointly explained 12.3% of the phenotypic variance (Table 1). Each of the 9 qSNPs was the most significant marker of the corresponding QTL peak shown in Figure 2B. The focus of our study was the detection of loci rather than the precise estimation of their effects. Previous work [2] has shown that grammar correction is effective at controlling type I error with little loss of power, but leads to underestimation of effects. Hence the full procedure requires the re-estimation of effects in a full mixed model. The latter was not performed in this study and so considering the use of GRAMMAR corrected trait, our results may have identified a considerable proportion of the actual phenotypic variance. The results from the three single-marker methods had high concordance (Figure 3), which suggested the final results to be fairly robust. Some difference in power of detection was found (Figure 4). However, in spite of the expectation that QTDT being more conservative, it does not seem to suffer from loss of power when many large full-sib families are available. QTDT may have the advantage of added protection against bias due to the unknown relatedness amongst the founders. On the other hand, QTDT is unable to handle the half-sib relationships that GRAMMAR can. Therefore, a combination of these approaches would compensate the shortcomings in each of the individual methods. In this case, they yielded very similar results. Thus, we favour the GRAMMAR method for its speed and flexibility.

**Table 1:** The final integrated mapping results using step-wise regression. The estimates of the allele substitution effect under the single additive QTL model are included for comparison.

SNP_1_	SNP_2 _^a^	logP^b^	"1/2" genotype^c^	"2/2" genotype^d^	Accumulated Variance (%)	Single QTL allelic effect (simulated)^g^
196	-	18.65	0.33	0.62	1.78	0.71 (0.62)
402	-	5.59	0.08	0.23	2.28	0.85 (0.56)
540^e^	3219	10.51 (7.75)	-0.18 (-0.09)	-0.30 (0.08)	3.53	
778	-	7.34	0.23	0.45	4.18	0.42 (0.37)
1257^f^	3689	9.15 (6.63)	-0.56 (-0.24)	0.46 (-0.17)	5.56	
1270	-	7.60	0.23	0.44	6.23	0.50 (0.35)
1483	-	10.02	-0.14	-0.36	7.13	0.43 (0.37)
2133	-	5.89	0.10	0.31	7.65	0.39 (0.30)
3033	-	20.52	0.31	0.66	9.54	0.68 (0.61)
3765	-	11.91	0.25	0.48	10.62	0.56 (0.58)
4935	-	18.38	-0.33	-0.71	12.35	0.70 (0.75)

^a^: SNP_2 _column has a value only for epistatic pairs.

^b^: the-LOG_10 _transformed P value for single significant SNPs, or the epistatic pairs in the nested test order. The -LOG_10 _(P) value for epistasis tests are in brackets.

^c^: estimate of the "1/2" genotype in contrast to the "1/1" genotype. The estimates of SNP_2 _are in brackets.

^d^: estimate of the "2/2" genotype in contrast to the "1/1" genotype. The estimates of SNP_2 _are in brackets.

^e^: estimates of the pair-wise interactions: 0.51, 0.23, 0.07 and 0.66 for "1/2 × 1/2", "2/2 × 1/2", "1/2 × 2/2" and "2/2 × 2/2", respectively.

^f^: estimates of the pair-wise interactions: 0.57, -0.55, 0.53 and -0.47 for "1/2 × 1/2", "2/2 × 1/2", "1/2 × 2/2" and "2/2 × 2/2", respectively.

^g^: Estimated allelic effect from the single SNP GRAMMAR model; the simulated value is taken from the nearest simulated QTL as provided by the organisers.

Bonferroni derived threshold adjust for the number of tests, but assume they are independent. As markers are correlated these thresholds will be too stringent and this will be particularly the case when dealing with pairs of correlated markers as is done in epistatic analyses. To explore to potential effects of this we relaxed each of the thresholds used in stages 2 to 4 of the epistatic analyses by 1 log *P *(equivalent to 10-fold fewer independent tests) and re-tested the interactions following the same algorithm. In addition to the two pairs detected, we listed 7 new pairs: 2 qSNP-qSNP (1271 & 4928, 1483 & 4942), 2 qSNP × non-qSNP (331 & 591, 991 & 3048) and 3 non-qSNP × non-qSNP (319 & 840, 1221 & 4555, 1564 & 3121). After integration with the 108 qSNPs, all pairs and 6 remaining qSNPs jointly explained about 15% of the phenotypic variance. Further effort is required to determine the appropriate thresholds for use in GWA epistasis analyses.

Using the ECDF computing facility , which is approximately three times faster compared to a standard desktop, the Merlin step of the QTDT software took 2 hours per chromosome while the actual QTDT analyses took about 4 hours per chromosome. A 'genome scan' using the single SNP GRAMMAR method took only 6 minutes on a standard desktop PC. Using the same machine, the 3-marker window analyses took 16 minutes, while the 3-marker haplotypes analyses took approximately 1 hour. The epistatic analyses for *n *SNPs require (*n*-1)*(*n*/2) calculations with additional terms to be estimated. These analyses ran for several days using a background queue at the ECDF facility therefore no reliable estimate of calculation time was available.

## Conclusion

Using several methods in analysing GWA can be useful in gaining confidence on the QTL identified. GRAMMAR is much faster to run than QTDT and takes into account complex relationships existing in general pedigrees. Furthermore, extending the model in GRAMMAR to study epistasis is reasonably easy and computationally feasible by using parallel or Grid computing.

## Epilogue

Following presentation of the simulation design, our strategy turned out to be effective and correctly detected 5 out of 6 major QTLs as well as 4 out of 45 smaller QTLs, despite the conservative thresholds employed in this study. The estimates of the genotypic QTL effects in the joint model (after stepwise regression) appear small compared to the simulated values. However, the estimates of the allelic QTL effects from the additive single marker GRAMMAR analyses compare quite well to the simulated values (Table 1). It seems that the joint fitting of the QTL reduces their estimates by > 50%. It was previously shown that the GRAMMAR approach leads to reduced estimates of the QTL effect [2] but that study did not account for the upward bias usually observed as a result of the 'Beavis effect'. Under a GRAMMAR approach it was recommended to re-estimate significant effect on the basis of the raw data using raw data and including a polygenic effect [2], which was not done in the present study. Only the present study modelled epistatic interaction between SNPs. Because no epistasis was actually simulated our results represent spurious effects. Overall, the strategy worked well but extensive simulations are required to derive appropriate thresholds, especially for detecting epistatic interactions.

## List of abbreviations used

GWA: Genome-Wide Association; QTL: Quantitative Trait Locus; MAF: minor allele frequency; SNP: Single Nucleotide Polymorphism; LD: Linkage Disequilibrium; GRAMMAR: Genome-wide Rapid Analysis using Mixed Models And Regression; QTDT: Quantitative Transmission Disequilibrium Test; qSNP: Quantitative SNP (SNP that is a putative QTL); logP: logarithm to the base 10 of the P value

## Competing interests

The authors declare that they have no competing interests.

## Authors' contributions

ACL, JP and WHW carried out the analyses and drafted the manuscript. DJK and CSH coordinated the analyses and the write-up. All authors have read and contributed to the final text of the manuscript.
